# PuMA: A papillomavirus genome annotation tool

**DOI:** 10.1093/ve/veaa068

**Published:** 2020-08-26

**Authors:** Josh Pace, Ken Youens-Clark, Cordell Freeman, Bonnie Hurwitz, Koenraad Van Doorslaer

**Affiliations:** School of Animal and Comparative Biomedical Sciences, University of Arizona, 1200 E. University Blvd. Tucson, AZ 85721-0073, USA; Department of Biomedical Engineering, University of Arizona, 1200 E. University Blvd. Tucson, AZ 85721-0073, USA; Department of Biosystems Engineering, University of Arizona, 1200 E. University Blvd. Tucson, AZ 85721-0073, USA; School of Animal and Comparative Biomedical Sciences, University of Arizona, 1200 E. University Blvd. Tucson, AZ 85721-0073, USA; Department of Electrical and Computer Engineering, University of Arizona, 1200 E. University Blvd. Tucson, AZ 85721-0073, USA; Department of Biosystems Engineering, University of Arizona, 1200 E. University Blvd. Tucson, AZ 85721-0073, USA; The BIO5 Institute, University of Arizona, 1200 E. University Blvd. Tucson, AZ 85721-0073, USA; School of Animal and Comparative Biomedical Sciences, University of Arizona, 1200 E. University Blvd. Tucson, AZ 85721-0073, USA; The BIO5 Institute, University of Arizona, 1200 E. University Blvd. Tucson, AZ 85721-0073, USA; Department of Immunobiology, Cancer Biology GIDP, Genetics GIDP, and UA Cancer Center, University of Arizona, 1200 E. University Blvd. Tucson, AZ 85721-0073, USA

**Keywords:** metagenomics, virome, annotation, papillomavirus, polyomavirus, high-throughput sequencing

## Abstract

High-throughput sequencing technologies provide unprecedented power to identify novel viruses from a wide variety of (environmental) samples. The field of ‘viral metagenomics’ has dramatically expanded our understanding of viral diversity. Viral metagenomic approaches imply that many novel viruses will not be described by researchers who are experts on (the genomic organization of) that virus family. We have developed the papillomavirus annotation tool (PuMA) to provide researchers with a convenient and reproducible method to annotate and report novel papillomaviruses. PuMA currently correctly annotates 99% of the papillomavirus genes when benchmarked against the 655 reference genomes in the papillomavirus episteme. Compared to another viral annotation pipeline, PuMA annotates more viral features while being more accurate. To demonstrate its general applicability, we also developed a preliminary version of PuMA that can annotate polyomaviruses. PuMA is available on GitHub (https://github.com/KVD-lab/puma) and through the iMicrobe online environment (https://www.imicrobe.us/#/apps/puma).

## 1. Introduction

Viruses and viral diseases have fascinated humans for millennia [reviewed in Woolhouse et al. (2012)]. We are currently in the era of ‘Neovirology’ (Enquist and Editors of the *Journal of Virology* 2009), and recent technological advances provide an agnostic approach to sequencing nucleotides from environmental samples. These approaches dramatically accelerated viral discovery (Hurwitz et al. 2016; Nooij et al. 2018). Excitingly, these ‘viromic’ approaches facilitate the identification of highly diverse viruses from a wide array of sample types. However, for these viromic studies to maximize their potential scientific impact, the identified viral genomes should be annotated and curated before publication. Many viruses are being identified by researchers who are not necessarily experts on that specific family of viruses, thus complicating a detailed curation process. Bioinformatic software packages have the potential to provide quick, accurate, and reproducible annotations. These software tools should be universally available, actively maintained, powerful yet intuitive, and automatable (Rose et al. 2016).

The *Papillomaviridae* (PVs) is a family of viruses with a circular, double-stranded DNA genome about 8,000 bp in length. Prototypical papillomavirus genomes contain six distinct open reading frames (ORFs). The early proteins (E1, E2, E6, E7) (Bergvall et al. 2013: 1; McBride 2013: 2; Roman and Munger 2013; Vande Pol and Klingelhutz 2013) manipulate the cellular environment to support viral replication, while the late L1 and L2 proteins are the viral structural proteins (Buck et al. 2013; Wang and Roden 2013). In addition, some viruses contain additional ORFs (e.g. E5, E8, E9, E10) (Van Doorslaer 2013). The viral upstream regulatory region (URR), is located downstream of the L1 ORF and contains binding sites for viral and host proteins (Bernard 2013).

We describe a papillomavirus annotation tool (PuMA). PuMA can be added to computational pipelines, or a graphical user interface is available through the iMicrobe online environment (Youens-Clark et al. 2019). When benchmarked against the papillomavirus episteme (PaVE) (Van Doorslaer et al. 2012, 2017), a database of manually annotated genomes, PuMA accurately annotates 99% of the virus genes present in PaVE, while annotating more features compared to other available pipelines. To demonstrate its potential broad usability, we adapted PuMA to annotate viruses belonging to a different taxonomic family.

## 2. Implementation

### 2.1 Philosophy

The papillomavirus annotation tool (PuMA) leverages a set of existing software tools and packages (see GitHub repository). The PuMA source code is written in Python 3 and extensively uses Biopython v1.72 (Cock et al. 2009) and other Python modules to handle and manipulate the data. Multiple sequence alignments are estimated using Muscle (Edgar 2004) and local database searches are performed using BLAST v2.7.1 (Camacho et al. 2009). Motif discovery and annotation are performed by MEME (Bailey and Elkan 1994) and FIMO (Grant et al. 2011), respectively. Detailed installation and usage instructions are provided on the PuMA github repository (https://puma-docs.readthedocs.io/en/latest/?badge=latest). PuMA is available under a broad GNU General Public License.

### 2.2 Reference genomes

Like many annotation pipelines, PuMA is extensively based on homology-based search approaches. Therefore, PuMA uses the manually curated sequences available in the PaVE database (Van Doorslaer et al. 2012, 2017). In March of 2020, the PuMA database contained 655 reference sequences with annotated features. As new genomes are added to PaVE, the PuMA database will also be updated. Conversely, PuMA annotations will be shared with PaVE to ensure maximum agreement between both resources.

### 2.3 Annotation of viral ORFs and putative proteins

In a first step, all stretches of at least seventy-five nucleotides located between two stop codons on the forward coding strand are translated into putative peptides. These putative peptides are used in a BLASTp homology (Altschul et al. 1990; Altschul et al. 2005) search against a custom protein database (available on the PuMA GitHub page) to identify the viral proteins and deduce ORFs.

This approach identifies the first in frame methionine as the start of the protein. However, in many cases (data not shown), this results in a faulty annotation. To identify the most likely start codon, a Blastp homology search is used to identify the closest neighbors of each newly identified protein. Based on *e*-value cutoffs (*e* > 1e−43), up to ten related proteins are identified. These eleven (putative protein and up to ten relatives) sequences are aligned using MUSCLE (Edgar 2004). Based on this alignment, the most parsimonious (i.e. shared by majority of proteins) is considered the conserved methionine start position.

Following identification of L1, the circular viral genome is linearized so that the first position following the stop codon for the L1 ORF is identified as position 1. This implies that all viral genomes will start with the URR.

### 2.4 Annotation of spliced viral transcripts and putative proteins

A typical papillomavirus combines several promoters and splice-donor and acceptor sites to transcribe up to twenty alternatively spliced mRNAs (Graham and Faizo 2017). At this time, PuMA automatically annotates the two most-studied alternatively spliced viral mRNAs, E1^E4 and E8^E2. The splice acceptor is shared between the E1^E4 and E8^E2 mRNAs and is embedded within the viral E2 ORF. We use MUSCLE (Edgar 2004) to align the newly annotated E2 ORF to its closest previously known relative. The splice acceptor site is identified based on the implied homology between both viruses. A similar homology-based approach is used to identify the splice donor sites for E1^E4 and E8^E2, both located within the E1 ORF (Puustusmaa and Abroi 2016).

### 2.5 Annotation of regulatory elements within the URR

The sequences for 655 known URR were downloaded from PaVE. These URR sequences were used in MEME (Bailey and Elkan 1994) to generate a consensus. For E1 binding sites, we used MEME to identify a single motif with length between eighteen and twenty-one bases (ATDATTGTTGNYAACAAYHAT; D: A, G, or T; N: any base; Y: C or T; H: A, C, or T). For the E2 binding sites, motifs of exactly 12 bp were identified (ACCGNWWNCGGT; W: A or T) binding site profile. For both motif searches, a custom background model, based on URR sequences in the training set was used. PuMA uses FIMO and these MEME generated profiles (Grant et al. 2011) to identify putative E1 or E2 binding sites within the viral URR (Figure 2A).

## 3. Running PuMA

### 3.1 Command-line

Briefly, PuMA is written in python3 and should run independently of operating systems. While PuMA has several dependencies, these are standard and are expected to be maintained by their respective developers. If these dependencies become deprecated, PuMA will endeavor to update its code to minimize disruptions. Detailed (installation) instructions are available on the PuMA GitHub repository. As a minimal requirement, PuMA requires a papillomavirus genome in FASTA format (e.g. new_PV.fa). Using the command-line interface, type:

python3 run_puma.py -i new_PV.fa

This will execute PuMA and generate different output files (Table 1).

**Table 1.** Output files provided by the PuMA annotation pipeline

File extension	Description
csv	A comma-separated file containing details for each putative annotation.
gff3	General Feature Formatted file containing details for each putative annotation.
Gb	NCBI GenBank formatted file containing details for each putative annotation.
fsa	Linearized genome in FastA format. This file can be used for GenBank submission.
tbl	NCBI tbl2asn TBL formatted file. This file can be used for GenBank submission.
pdf	Graphical representation of the newly annotated viral genome.

To minimize the need to download, install, and maintain different dependencies PuMA is also available from Docker Hub. These Docker ‘containers’ are isolated from the local environment on a user computer and come pre-packaged with all the software installed, ready for use. Docker images can be downloaded from the PuMA GitHub page.

### 3.2. iMicrobe

**Figure 1.**
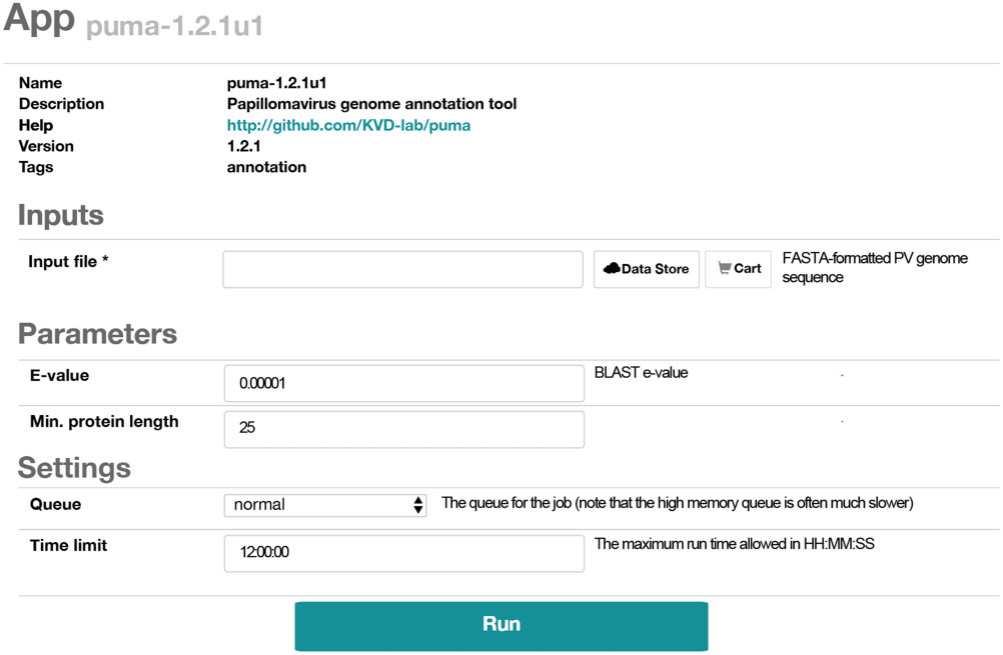
Screenshot of PuMA submission form on iMicrobe.

iMicrobe (Youens-Clark et al. 2019) provides a graphical user interface to run PuMA. Login to your free account and navigate to ‘my account’. Use the *Data Store menu* to upload your data to iMicrobe. Navigate to the PuMA app within iMicrobe and use the ‘Data Store’ button to select your genome file (Figure 1). We recommend running PuMA using the default settings. This analysis will provide the same data as obtained through the command-line settings (Table 1). Data can be downloaded for offline use. iMicrobe accesses the Extreme Science and Engineering Discovery Environment compute resources at Texas Advanced Computing Center including Stampede2. While this offers access to significant compute power, the large number of users means that a job might be in the queue for hours. Once the job runs, it takes a few seconds for execution to complete.

### 3.3 Output files

PuMA generates several output files. The initial folder is named according to the virus name. This folder contains two distinct folders. The ‘program_files’ folder has all the files that PuMA generated while running. These can be used for troubleshooting purposes, but do not contain final results, and can typically be ignored. The ‘for_user’ folder contains the annotated viral genome in several distinct formats (Table 1), as well as a log file describing the progress of the analysis. In addition to several annotated genome files, PuMA also provides the input files needed to streamline submission to GenBank using the NCBI tool tbl2asn (https://www.ncbi.nlm.nih.gov/genbank/tbl2asn2/). If the user wants to use PuMA to submit to GenBank, an additional template file containing meta-data is needed. This file can be generated through the NCBI (https://submit.ncbi.nlm.nih.gov/genbank/template/submission/).

## 4. PuMA evaluation

### 4.1 Dropout testing

PuMA is built around homology-based approaches. Therefore, to compare the accuracy of PuMA to the manually annotated PaVE genomes, we performed dropout testing using all genomes in the PaVE database. Practically, we ran PuMA as described above but use custom databases excluding the test genome and associated annotations. This way, the test genome shares at most 90% sequence identity with its closest neighbor in the database (Bernard et al. 2010). We automated this process (available on GitHub) and annotated all 655 genomes on the PaVE database. Following annotation, the PuMA output was compared to the annotations currently on PaVE. Python scripts to replicate these analyses are included in the PuMA GitHub repository.

### 4.2 Comparison of PuMA to viral annotation pipeline and identification

We ran viral annotation pipeline and identification (VAPiD) (Shean et al. 2019) using default settings. To ensure a fair comparison between PuMA and VAPiD, we ensured that VAPiD used a local database consisting of the manually curated PaVE genomes, and not the default NCBI GenBank database. We utilized the dropout tests as described for the testing of PuMA above. Following annotation, the PuMA output was compared to the annotations currently on PaVE. Python scripts to replicate these analyses are included in the PuMA GitHub repository. We acknowledge that VAPiD’s annotation power is derived from identifying closely related viruses and transferring the annotation. As such, our defined database of PaVE genomes may have lowered the accuracy of VAPiD.

## 5. Results and discussion

**Figure 2.**
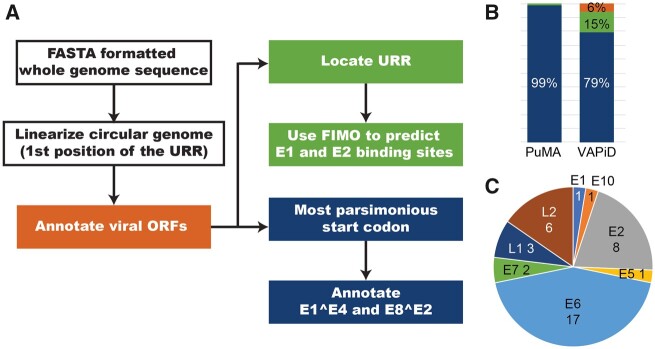
(A) Flowchart illustrating the PuMA algorithm: see the methods section for more details. (B) PuMA correctly annotates 99% (blue) of manual annotations present on the PaVE database. PuMA annotations were compared to the manually curated genomes in the PaVE database. The previously described VAPiD algorithm correctly identifies 79% of all annotations, wrongly annotates 15%, and did not identify 6% of possible annotations (C) proteins that were wrongly identified by PuMA v1.2.1 are plotted.

We developed PuMA to facilitate the consistent and uniformly annotation of papillomavirus genomes. To test the performance of PuMA, we downloaded 655 FASTA-formatted viral genomes (with 6,557 associated annotations) from PaVE [(Van Doorslaer et al. 2012, 2017); accessed on 15 January 2020]. These genomes were manually annotated by experts in papillomavirus biology (Van Doorslaer et al. 2012, 2017). These PaVE reference genomes were used for the development and testing of PuMA. PuMA uses a FASTA formatted sequence of a novel papillomavirus genome, linearizes the circular genome, and annotates putative proteins and regulatory elements (Figure 2). The output from PuMA can be used in downstream applications or uploaded to GenBank using the Sequin submission tool. To test the accuracy of PuMA, we used a drop-out test to compare PuMA to the manually annotated features in PaVE database. Using this approach, the current version of PuMA (version 1.2.1) accurately annotates ∼99% of the genes in PaVE (Figure 2B). Without additional information, PuMA will annotate the longest putative protein in each ORF (starting at the first methionine). This does not always identify the correct start position. To address this, PuMA uses a multiple sequence alignment with homologous sequences to identify the most parsimonious start position for each gene. Nonetheless, all but one of the discrepancies between PuMA and the (manually) annotated genomes are due to differences in start codon prediction. Alignments comparing the PuMA annotation to PaVE are available on the PuMA GitHub page.

The homology-based approach used by PuMA assumes that the manually annotated sequences on PaVE are correct and biologically relevant. However, since the majority of the viral proteins have not been experimentally validated, it is difficult to confirm the biological accuracy of these annotations. This is highlighted by the prediction of the E1^E4 and E8^E2 spliced transcripts. Splice site prediction is a notoriously hard problem, and this is not different for papillomavirus genes. Out of 1269 E1^E4 and E8^E2 annotations in PaVE, PuMA and PaVE disagree on 159. However, in the absence of biological validation, it is impossible to know which annotation is correct. To improve the performance of PuMA while maximizing the accuracy of the PaVE database, there will be continuous exchange of database annotations between PuMA and PaVE. As more biological data becomes available, this will be incorporated in PaVE and PuMA.

Other pipelines for annotating viral genomes have been described. Specifically, the recently described VAPiD (Shean et al. 2019) pipeline has many similarities with PuMA. Unlike PuMA, VAPiD was developed to annotate many different viral families. To provide a general idea on PuMA’s performance, we compare PuMA to VAPiD. Both programs rely on blast-based homology searches. To allow for a fair comparison, both programs used the manually annotated PaVE reference genomes as the base of their annotations. Overall, PuMA identifies more papillomavirus genes (correctly) compared to VAPiD (Figure 2B). Also, PuMA identifies spliced transcripts and regulatory elements that are not identified by VAPiD. PuMA was created as a papillomavirus specific tool, while VAPiD annotates very diverse viral families. Therefore, it is to be expected that PuMA is more accurate. This in no means devaluates VAPiD as a powerful annotation pipeline. However, if papillomavirus genome annotation is the goal, PuMA should be used. PuMA derives its power from the manually annotated annotations on PaVE.

We will continue to incorporate new biological information to improve PuMA’s annotation accuracy. For example, the L1 protein for the recently described fish papillomavirus, SaPV1, (López-Bueno et al. 2016) is likely encoded from a spliced mRNA. However, since this a unique feature for this virus, PuMA does not annotate this spliced mRNA, but wrongly annotates a short L1 based on the canonical (mammalian) papillomavirus genomes. Once more viruses show these unique features, PuMA will be updated to provide accurate annotations of these fish viruses.

Since PuMA relies on homology-based searches to known viruses, it is easy to adapt PuMA to annotate different viral families. To demonstrate this, we modified PuMA to annotate polyomavirus genomes. Expertly annotated viral genomes were downloaded from PyVE (http://home.ccr.cancer.gov/lco/PyVE.asp). We updated the primary BLAST database to contain polyomavirus sequences (available on GitHub). Since polyomaviruses are translated bi-directionally, we edited the PuMA code to annotate both the forward and reverse strands of the polyomavirus genomes. Without making additional changes, PuMA correctly annotates 87% of polyomavirus proteins compared to the manually annotated PyVE derived genomes. While this accuracy can be improved, this exercise demonstrates that, given a well-annotated database, PuMA can be used to annotate a wide array of viruses belonging to different families.

## 6. Conclusion

The goal of PuMA is to provide users with an automated means of uniform and unbiased papillomavirus genome annotation. With the development of new sequencing technologies, PuMA will aid in the labor-intensive process of annotating the provided genome. Even though PuMA is papillomavirus specific, the open-source software combined with the custom functions allows for easy adaption for other viruses by merely changing the background databases. This was demonstrated by adapting PuMA to annotate a different family of viruses.

## References

[veaa068-B1] Altschul S. F. et al (1990) ‘Basic Local Alignment Search Tool’, Journal of Molecular Biology, 215: 403–10. 2231712 10.1016/S0022-2836(05)80360-2

[veaa068-B2] Altschul S. F. et al (2005) ‘Protein Database Searches Using Compositionally Adjusted Substitution Matrices’, FEBS Journal, 272: 5101–9. 16218944 10.1111/j.1742-4658.2005.04945.x PMC1343503

[veaa068-B3] Bailey T. L. Elkan C. (1994) ‘Fitting a Mixture Model by Expectation Maximization to Discover Motifs in Biopolymers’, Proceedings International Conference on Intelligent Systems for Molecular Biology, 2: 28–36. 7584402

[veaa068-B4] Bergvall M. Melendy T. Archambault J. (2013) ‘The E1 Proteins’, Virology, 445: 35–56. 24029589 10.1016/j.virol.2013.07.020 PMC3811109

[veaa068-B5] Bernard H.-U. et al (2010) ‘Classification of Papillomaviruses (PVs) Based on 189 PV Types and Proposal of Taxonomic Amendments’, Virology, 401: 70–9. 20206957 10.1016/j.virol.2010.02.002 PMC3400342

[veaa068-B6] Bernard H.-U. (2013) ‘Regulatory Elements in the Viral Genome’, Virology, 445: 197–204. 23725692 10.1016/j.virol.2013.04.035

[veaa068-B7] Buck C. B. Day P. M. Trus B. L. (2013) ‘The Papillomavirus Major Capsid Protein L1’, Virology, 445: 169–74. 23800545 10.1016/j.virol.2013.05.038 PMC3783536

[veaa068-B8] Camacho C. et al (2009) ‘BLAST+: Architecture and Applications’, BMC Bioinformatics, 10: 421. 20003500 10.1186/1471-2105-10-421 PMC2803857

[veaa068-B9] Cock P. J. A. et al (2009) ‘Biopython: Freely Available Python Tools for Computational Molecular Biology and Bioinformatics’, Bioinformatics, 25: 1422–3. 19304878 10.1093/bioinformatics/btp163 PMC2682512

[veaa068-B10] Edgar R. C. (2004) ‘MUSCLE: Multiple Sequence Alignment with High Accuracy and High Throughput’, Nucleic Acids Research, 32: 1792–7. 15034147 10.1093/nar/gkh340 PMC390337

[veaa068-B11] Enquist L. W. ; Ediotrs of the Journal of Virology (2009) ‘Virology in the 21st Century’, Journal of Virology, 83: 5296–308. 19297504 10.1128/JVI.00151-09 PMC2681991

[veaa068-B12] Graham S. V. Faizo A. A. A. (2017) ‘Control of Human Papillomavirus Gene Expression by Alternative Splicing’, Virus Research, 231: 83–95. 27867028 10.1016/j.virusres.2016.11.016 PMC5335905

[veaa068-B13] Grant C. E. Bailey T. L. Noble W. S. (2011) ‘FIMO: Scanning for Occurrences of a Given Motif’, Bioinformatics, 27: 1017–8. 21330290 10.1093/bioinformatics/btr064 PMC3065696

[veaa068-B14] Hurwitz B. L. U’Ren J. M. Youens-Clark K. (2016) ‘Computational Prospecting the Great Viral Unknown’, FEMS Microbiology Letters, 363: fnw077. 27030726 10.1093/femsle/fnw077

[veaa068-B15] López-Bueno A. et al (2016) ‘Concurrence of Iridovirus, Polyomavirus, and a Unique Member of a New Group of Fish Papillomaviruses in Lymphocystis Disease-Affected Gilthead Sea Bream’, Journal of Virology, 90: 8768–79. 27440877 10.1128/JVI.01369-16 PMC5021401

[veaa068-B16] McBride A. A. (2013) ‘The Papillomavirus E2 Proteins’, Virology, 445: 57–79. 23849793 10.1016/j.virol.2013.06.006 PMC3783563

[veaa068-B17] Nooij S. et al (2018) ‘Overview of Virus Metagenomic Classification Methods and Their Biological Applications’, Frontiers in Microbiology, 9: 749. 29740407 10.3389/fmicb.2018.00749 PMC5924777

[veaa068-B18] Puustusmaa M. Abroi A. (2016) ‘Conservation of the E8 CDS of the E8^E2 Protein Among Mammalian Papillomaviruses’, Journal of General Virology, 97: 2333–45. 27325292 10.1099/jgv.0.000526

[veaa068-B19] Roman A. Munger K. (2013) ‘The Papillomavirus E7 Proteins’, Virology, 445: 138–68. 23731972 10.1016/j.virol.2013.04.013 PMC3783579

[veaa068-B20] Rose R. et al (2016) ‘Challenges in the Analysis of Viral Metagenomes’, Virus Evolution, 2: vew022. 29492275 10.1093/ve/vew022 PMC5822887

[veaa068-B21] Shean R. C. et al (2019) ‘VAPiD: A Lightweight Cross-Platform Viral Annotation Pipeline and Identification Tool to Facilitate Virus Genome Submissions to NCBI GenBank’, BMC Bioinformatics, 20: 48. 30674273 10.1186/s12859-019-2606-y PMC6343335

[veaa068-B22] Van Doorslaer K. (2013) ‘Evolution of the Papillomaviridae’, Virology, 445: 11–20. 23769415 10.1016/j.virol.2013.05.012

[veaa068-B23] Van Doorslaer K. et al (2012) ‘The Papillomavirus Episteme: A Central Resource for Papillomavirus Sequence Data and Analysis’, Nucleic Acids Research, 41: D571–8. 23093593 10.1093/nar/gks984 PMC3531071

[veaa068-B24] Van Doorslaer K. et al (2017) ‘The Papillomavirus Episteme: A Major Update to the Papillomavirus Sequence Database’, Nucleic Acids Research, 45: D499–506. 28053164 10.1093/nar/gkw879 PMC5210616

[veaa068-B25] Vande Pol S. B. Klingelhutz A. J. (2013) ‘Papillomavirus E6 Oncoproteins’, Virology, 445: 115–37. 23711382 10.1016/j.virol.2013.04.026 PMC3783570

[veaa068-B26] Wang J. W. Roden R. B. S. (2013) ‘L2, the Minor Capsid Protein of Papillomavirus’, Virology, 445: 175–86. 23689062 10.1016/j.virol.2013.04.017 PMC3770800

[veaa068-B27] Woolhouse M. et al (2012) ‘Human Viruses: Discovery and Emergence’, Philosophical Transactions of the Royal Society B: Biological Sciences, 367: 2864–71. 10.1098/rstb.2011.0354 PMC3427559 22966141

[veaa068-B28] Youens-Clark K. et al (2019) ‘iMicrobe: Tools and Data-Driven Discovery Platform for the Microbiome Sciences’, GigaScience, 8: giz097 31372655 10.1093/gigascience/giz097 PMC6675616

